# Application of diagnostic criteria in paediatric complex regional pain syndrome: a scoping review protocol

**DOI:** 10.1136/bmjopen-2025-101963

**Published:** 2025-05-16

**Authors:** See Wan Tham, Rebecca Lee, Lisa-Marie Rau, Navil Firoze Sethna, Elisabeth Mueller Nylander, Lorenzo Fabrizi, Julia Wager, Helen Koechlin

**Affiliations:** 1 Department of Anesthesiology and Pain Medicine, University of Washington School of Medicine, Seattle, Washington, USA; 2 Center for Child Health, Behavior and Development, Seattle Children’s Research Institute, Seattle, Washington, USA; 3 Center for Epidemiology, Center for Musculoskeletal Research, Division of Musculoskeletal and Dermatological Sciences, The University of Manchester Faculty of Biology Medicine and Health, Manchester, UK; 4 National Institute for Health Research Biomedical Research Center, Manchester University NHS Foundation Trust, Manchester, UK; 5 Manchester Centre for Health Psychology, Division of Psychology and Mental Health, The University of Manchester, Manchester, UK; 6 German Paediatric Pain Centre, Children’s and Adolescents’ Hospital Datteln, Datteln, Germany; 7 Department of Children’s Pain Therapy and Paediatric Palliative Care, Faculty of Health, School of Medicine, Witten/Herdecke University, Witten, Germany; 8 Anesthesiology, Critical Care and Pain Medicine, Harvard Medical School, Boston Children’s Hospital, Boston, Massachusetts, USA; 9 Library and Information Commons, Seattle Children’s Research Center, Seattle Children’s Hospital, Seattle, Washington, USA; 10 Department of Neuroscience, Physiology and Pharmacology, Division of Biosciences, University College London, London, UK; 11 German Paediatric Pain Centre, Children's and Adolescents’ Hospital Datteln, Datteln, Germany; 12 Department of Children’s Pain Therapy and Paediatric Palliative Care, Faculty of Health, School of Medicine, Universitat Witten/Herdecke, Witten, Germany; 13 Psychosomatics and Psychiatry, University Children’s Hospital Zürich, Zürich, Switzerland; 14 Child and Adolescent Health Psychology, University of Zurich Department of Psychology, Zürich, Switzerland; 15 Children’s Research Center, University Children’s Hospital Zürich, Zürich, Switzerland

**Keywords:** Child, Adolescent, Chronic Pain

## Abstract

**Abstract:**

**Introduction:**

There are no validated paediatric-specific diagnostic criteria for complex regional pain syndrome (CRPS). As a result, diagnostic tools developed for adults (eg, Budapest Criteria, Japanese Diagnostic Criteria, Veldman Criteria) are frequently applied in the paediatric population. However, the clinical presentations and trajectories of children can differ from adults. Given that treatment outcomes are linked to early diagnosis and intervention, the lack of paediatric-specific screening or diagnostic tools is an important knowledge gap. We aim to identify the frequency of individual criteria used in diagnosing CRPS in children and adolescents in existing literature, summarise assessment methods used to establish the diagnosis, and provide recommendations for research and clinical application.

**Methods:**

The following databases and platforms will be searched for articles published from 2003 (year the Budapest Criteria was developed) onward: CINAHL, CENTRAL, Embase, Ovid MEDLINE, PubMed, PsycINFO and Web of Science. Our search strategy will use subject headings and text words related to the concepts of CRPS in paediatric populations, with study inclusion criteria from birth up to 18 years old, and a diagnosis of CRPS. Data will be extracted by our multidisciplinary team and findings will be reported in accordance with the Preferred Reporting Items for Systematic Reviews and Meta-Analyses extension for Scoping Reviews.

**Ethics and dissemination:**

This study does not involve human participants or unpublished data; therefore, approval from a human research ethics committee is not required. The findings of this scoping review will be disseminated through academic conferences and peer-reviewed publications.

**STRENGTHS AND LIMITATIONS OF THIS STUDY**

- This scoping review will systematically identify the frequency of criteria used in diagnosing paediatric complex regional pain syndrome (CRPS), thereby addressing an important gap in the literature.
- Through an analysis of applied diagnostic criteria, this scoping review will provide suggestions for paediatric specifications for CRPS and recommendations for clinical practice and implementation.
- Findings on the frequency of diagnostic criteria for CRPS in children and adolescents are expected to be heterogeneous. However, this will provide a foundation for developing a systematic approach to standardise diagnosis and facilitate further psychometric evaluation.

## Introduction

 Complex regional pain syndrome (CRPS) is a chronic pain condition that presents with severe pain, affecting the distal extremities.^1^ This is accompanied by altered sensory perception such as hyperalgesia and/or allodynia, vasomotor, trophic and autonomic dysfunction.^2 3^ There may be an inciting event (eg, trauma); however, no causes may be identified in paediatric populations.^4^ The clinical presentation of debilitating pain, skin discolouration, temperature changes, swelling and hyperhidrosis of the affected region is highly distressing for children and adolescents, leading to significant pain-related disability.^5^ The incidence of paediatric CRPS is estimated to be 1.14/100 000 children per year.^6^ The underlying pathophysiology remains poorly understood, and it is hypothesised that dysfunction within the somatosensory system, combined with a complex interplay of biopsychosocial risk factors, contributes to its manifestation.^7^,^10^

There are challenges in formulating the diagnosis of CRPS, as there are no confirmatory objective test(s) or biological markers. In adults, validated diagnostic criteria have been developed, such as the Budapest Criteria,^11^ which was formally adopted by the International Association for the Study of Pain in 2003 following a consensus conference in Budapest. Those criteria have since been identified as the gold standard for diagnosis of CRPS in adults.^4^ Unfortunately, research remains nascent on the development and validation of diagnostic criteria in paediatric populations. Given this limitation, adult diagnostic criteria have been applied clinically and in research studies for children.^12^ However, based on age and developmental stage, the transferability is questionable as clinical characteristics, symptom trajectory and response to treatment in children differ from adults.^6 13 14^ For example, involvement of the lower extremities is more common in children, and trophic changes are similarly uncommon.^13^ Furthermore, studies also suggest that symptoms in children are milder, and the longitudinal trajectory seems more favourable compared with adults.^11^ Recognising that the developmental, neurocognitive and behavioural differences can also impact clinical presentation and evaluation, the direct application of adult diagnostic criteria for children may inadvertently result in delayed or inaccurate diagnoses and inappropriate care. As treatment outcomes (eg, physical functioning, pain severity) have been linked to early diagnosis and intervention,^13^ the lack of paediatric-specific screening or diagnostic tools for CRPS is a significant research and knowledge gap.

Given the lack of paediatric-specific diagnostic tools, the Budapest Criteria is commonly used to diagnose CRPS in paediatric populations.^15 16^ However, in findings from a systematic review on paediatric CRPS, the majority of studies (>50%) used authors’ own criteria and/or assigned the diagnosis based upon clinician expertise, further highlighting this research gap in diagnostic consistency.^15^ More recently, research has advanced in the work by Mesaroli and colleagues, who developed a paediatric screening tool, the Pediatric PainSCAN. Preliminary validation efforts focused on establishing content validity for paediatric CRPS and neuropathic pain.^17^ This measure has yet to undergo additional psychometric testing, limiting its application to date.

Besides formulating paediatric-specific criteria, methods of clinical assessment for identifying each criterion within the diagnostic tool should be considered.^18^ The process of obtaining a history and physical examination to evaluate for the presence of clinical features can differ dramatically between children and adults, as would be expected given the age-related developmental, cognitive, behavioural and communication considerations. Moreover, children’s neurological and physiological development undergo constant changes across the paediatric lifespan, lending to potential differences within the paediatric age groups.^19^ A paediatric-focused tool should incorporate these multidimensional factors in order to address these differences.

Taken together, there is a need to identify the diagnostic criteria used for diagnosing CRPS in paediatric populations and to systematically characterise the criteria that have been used in previous studies, including authors’ own criteria and clinician assessments. Therefore, the objectives of this scoping review are to identify the frequency of individual criteria used in diagnosing CRPS in children and summarise the assessment methods used to establish the diagnosis. Finally, based on our results, we will provide recommendations for the use of the diagnostic criteria in paediatric clinical practice and research.

## Methods and analysis

This proposed scoping review will be reported in accordance with the Preferred Reporting Items for Systematic Reviews and Meta-Analyses extension for Scoping Reviews (PRISMA-ScR) guidelines and is registered with the Open Science Framework (OSF: https://osf.io/j8mp3/).

### Eligibility criteria

#### Participants

We are interested in studies on participants ages birth up to 18 years old, with a clinical diagnosis of CRPS. Studies should include any type of established diagnostic criteria (eg, Budapest Criteria, Veldman Criteria) and those as defined by authors (eg, symptoms, signs).

If studies include samples that span a larger age range into early adulthood, for example, 14–21 years, they can be included, when either the mean age is below 18 years, with a maximum age of <24 years, as previously recommended for paediatric pain research,^20^ or outcomes are reported separately for patients ≤18 years.^21^

#### Types of evidence sources

This scoping review will include published peer-reviewed full text articles written in English, presenting original data with retrospective or prospective study designs, including case reports, case series, case–control, cross-sectional, time-series, longitudinal cohort and randomised controlled trials studies. Commentaries may be included if the inclusion criteria are met and original data are presented. The following types of articles will be excluded: reviews (eg, narrative, scoping, systematic), meta-analyses, clinical trial registrations, letters to the editor, opinion articles, essays, dissertations, conference abstracts, posters, books, book reviews and book chapters.

### Search strategy

Before undertaking this review, the PROSPERO database was searched for ongoing or recently completed systematic reviews and/or meta-analyses on the same topic. A research librarian (EN) with expertise in evidence synthesis will develop search strategies to reflect the concepts outlined in table 1 below. A PubMed search strategy was developed with input from the project team, and then peer reviewed by a second librarian, not otherwise associated with the project. The intended PubMed search strategy is included below (online supplemental appendix 1). This search strategy will be adapted to the syntax of the other databases/platforms, using search words for the title and abstract fields and controlled vocabulary whenever possible.

**Table 1:** Concepts and key terms for search blocks

MeSH	**“**pediatrics**”**[MeSH]**“**infant**”**[MeSH]**“**child**”**[MeSH]**“**child, hospitalized**”**[MeSH]**“**adolescent**”**[MeSH]**“**adolescent hospitalized**”**[MeSH]**“**minors**”**[MeSH]**“**young adult**”**[MeSH]	“complex regional pain syndromes**”**[MeSH]
synonyms	pediatric*adolescentboychildgirljuvenilekidminorpubescentpreadolescenceteenyoung adultyouth	algodystrophic syndromealgodystrophyalgoneurodystrophycausalgiachronic regional pain syndromecomplex regional pain syndromeCRPSMorbus Sudeckpost-traumatic dystrophyreflex sympathetic dystrophysympathetic dystrophysympathetic reflex dystrophySudeck’s atrophy/disease/syndromeSudeck Leriche syndrometype 1 regional pain syndrome

* Plural forms and alternate spellings will be included, for example, pediatric and pediatrics.

The following databases/platforms will be searched: APA PsycINFO (EBSCOHost), CINAHL (EBSCOHost), Cochrane Central Register of Controlled Trials (Wiley), Embase.com, Ovid MEDLINE, PubMed (PubMed-Not-Medline and PubMed In-Process), and Web of Science (WOS, MEDLINE, SCIELO). Searches will be limited to human studies and published from 2003 onward, because the most established CPRS criteria, the Budapest Criteria, were introduced at this time. As relevant studies are identified, we will check for additional relevant cited and citing articles.

### Data management and study selection

Search results will be exported into Covidence, a web-based software by Cochrane to assist with the production of reviews (https://www.covidence.org), to enable the identification and removal of duplicates. Study selection will be based on the previously described inclusion/exclusion criteria. For each record, two team members will independently perform the screening process on the title/abstract level as well as the full text assessment. Any disagreements during the screening process will be resolved by a third reviewer, and, if necessary, through discussions in the research team.

### Data extraction plan

A customised, standardised extraction form will be used, and data will be organised in a customised extraction form specifically structured for this scoping review. We will extract information on authors, year of publication, countries of study conduct, study aims, study design, sample size, measures for diagnosis of CRPS, individual diagnostic criteria for CRPS, additional symptoms and signs related to CRPS but not part of diagnostic criteria defined in measures, assessment procedures (eg, history vs examination; type of procedure; specific instructions on the evaluation process and interpretations rules), measures assessing psychosocial functioning (eg, of anxiety, depression, post-traumatic stress disorder, critical life events). Reviewers will be trained on using the data extraction form, and we will pilot the data extraction form on a sample of studies to ensure a common understanding among the reviewers. Next, independent data extraction of five randomly selected publications will be conducted by at least two reviewers, followed by discussion of disagreements among the entire team. After reaching agreement, subsequent data extraction for each study will be conducted by one team member. The reliability of extraction will be reviewed by a second team member throughout the extraction process, through regular checks to validate the extracted data.

### Data synthesis

A search decision flow chart will present the number of studies identified, excluded and included in accordance with the PRISMA-ScR guidance. Data will be summarised and analysed descriptively, and study characteristics will be presented in tabulated format. We will determine the frequency of using measures for the diagnosis of CRPS, individual diagnostic criteria for CRPS, additional symptoms and signs related to CRPS (but not part of diagnostic criteria), and the inclusion of measures that assessed psychosocial functioning. Based on this quantification, we will present the most prominent signs/symptoms across diagnostic tools/criteria.

## Ethics and dissemination

This study does not involve human participants or unpublished secondary data. Approval from a human research ethics committee is not required. The results of this scoping review will be disseminated through peer-reviewed publications, academic conferences and professional societies.

### Patient and public involvement

While patients and the public were not directly involved in the initial development of the scoping review protocol design, we plan to engage relevant patient representatives (eg, children, caretakers) and advocacy groups (eg, International Association for the Study of Pain, Complex Regional Pain Syndrome Special Interest Group) during the interpretation of results and dissemination phase. Future engagement strategies may include co-produced dissemination materials to ensure that the outcomes of this review are meaningful and applicable to the medical and patient communities.

## Supplementary material

10.1136/bmjopen-2025-101963 online supplemental appendix 1
