# Supplementary material for: Application of diagnostic criteria in paediatric complex regional pain syndrome: a scoping review protocol
Source: BMJ Open. 2025 May 16;15(5):e101963. doi: 10.1136/bmjopen-2025-101963 (PMC12086909; doi:10.1136/bmjopen-2025-101963)
Supplement: online supplemental appendix 1 [file bmjopen-15-5-s001.docx]

**Appendix 1**

Example PubMed search strategy

("pediatrics"[MeSH Terms] OR "child"[MeSH Terms] OR "child, hospitalized"[MeSH Terms] OR "adolescent"[MeSH Terms] OR "adolescent, hospitalized"[MeSH Terms] OR "minors"[MeSH Terms] OR "young adult"[MeSH Terms] OR "paediatric*"[Title/Abstract] OR "pediatric*"[Title/Abstract] OR "adolescen*"[Title/Abstract] OR "boy"[Title/Abstract] OR "boys*"[Title/Abstract] OR "child*"[Title/Abstract] OR "children*"[Title/Abstract] OR "girl*"[Title/Abstract] OR "juvenile*"[Title/Abstract] OR "kid"[Title/Abstract] OR "kids*"[Title/Abstract] OR "minor*"[Title/Abstract] OR "minors*"[Title/Abstract] OR "pre adolescen*"[Title/Abstract] OR "preadolescen*"[Title/Abstract] OR "preteen*"[Title/Abstract] OR "pubescen*"[Title/Abstract] OR "teen*"[Title/Abstract] OR "teenage*"[Title/Abstract] OR "young adult*"[Title/Abstract] OR "young individual*"[Title/Abstract] OR "young man"[Title/Abstract] OR "young men"[Title/Abstract] OR "young person*"[Title/Abstract] OR "young people*"[Title/Abstract] OR "young woman*"[Title/Abstract] OR "young women*"[Title/Abstract] OR "youngster*"[Title/Abstract] OR "youth*"[Title/Abstract]) AND ("complex regional pain syndromes"[MeSH Terms] OR "algodystroph*"[Title/Abstract] OR "algoneurodystroph*"[Title/Abstract] OR "causalgia*"[Title/Abstract] OR "chronic regional pain syndrome*"[Title/Abstract] OR "complex regional pain syndrome*"[Title/Abstract] OR "crps*"[Title/Abstract] OR "morbus sudeck"[Title/Abstract] OR "post traumatic dystroph*"[Title/Abstract] OR "posttraumatic dystroph*"[Title/Abstract] OR "reflex sympathetic dystroph*"[Title/Abstract] OR "sudeck* atroph*"[Title/Abstract] OR "sudeck* disease"[Title/Abstract] OR "sudeck* dystroph*"[Title/Abstract] OR "sudeck leriche syndrome*"[Title/Abstract] OR "sudeck* syndrome*"[Title/Abstract] OR "sympathetic dystroph*"[Title/Abstract] OR "sympathetic reflex dystroph*"[Title/Abstract]) AND 2003/01/01:2024/12/31[Date - Publication] AND "english"[Language]
