# Supplementary material for: Application of diagnostic criteria in paediatric complex regional pain syndrome: a scoping review protocol
Source: BMJ Open. 2025 May 16;15(5):e101963. doi: 10.1136/bmjopen-2025-101963 (PMC12086909; doi:10.1136/bmjopen-2025-101963)
Supplement: Supplementary file 2 [file bmjopen-2025-101963.reviewer_comments.pdf]

PEER REVIEW HISTORY

BMJ Open publishes all reviews undertaken for accepted manuscripts. Reviewers are asked to complete a checklist review form and are provided with free text boxes to elaborate on their assessment. These free text comments are reproduced below.

ARTICLE DETAILS

Title (Provisional)

Application of diagnostic criteria in pediatric complex regional pain syndrome: A scoping review protocol

Authors

Tham, See Wan; Lee, Rebecca; Rau, Lisa-Marie; Sethna, Navil Firoze; Nylander, Elisabeth Mueller; Fabrizi, Lorenzo; Wager, Julia; Koechlin, Helen

---

VERSION 1 - REVIEW

**Reviewer**	**1**
**Name**	**Jones, Abigail**
**Affiliation**	**University of the West of England, Psychology**
**Date**	**24-Mar-2025**
**COI**	**None**

This is an excellent protocol for a much-needed review and I have no suggested revisions. I look forward to reading the completed review in time.

**Reviewer**	**2**
**Name**	**Coventry, Jessica**
**Affiliation**	**Monash University**
**Date**	**02-Apr-2025**
**COI**	**None**

Thank you for this clear protocol. This is an important step in improving pediatric care for complex regional pain syndrome. I look forward to reading your results once the paper is published.

**Reviewer**	**3**
**Name**	**Riiser, Kirsti**

Affiliation **Oslo Metropolitan University, Rehabilitation Science and Health Technology**

Date **06-Apr-2025**

COI **None**

Thank you for the opportunity to peer-review this protocol. You make a strong case for the importance of conducting this scoping review. The protocol appears to be well-prepared and well-structured. I only have a few comments.

Introduction

-Line 37, Page 7: «Evaluated» should be written «evaluate»

-Line 5, page 8: The authors should consider rephrasing the third aim. As it stands now, it appears that the authors already make inferences based on the findings from the review.

-Line 12, page 8: «This proposed scoping review is reported in accordance with the PRISMA-ScR guidelines» should be rewritten to «The proposed scoping review will be reported in accordance with the PRISMA-ScR guidelines».

Methods

-If registered (e.g. in OSF), please provide the name of the registry and the registration number.

-Line 18, page 8: The authors should provide a reference for limiting the pediatric population to up to 18 years.

VERSION 1 - AUTHOR RESPONSE

Response to Reviewers

Reviewer #1:

This is an excellent protocol for a much-needed review and I have no suggested revisions. I look forward to reading the completed review in time.

Reviewer #2:

Thank you for this clear protocol. This is an important step in improving pediatric care for complex regional pain syndrome. I look forward to reading your results once the paper is published.

- *We appreciate the comments from Reviewers #1 and #2, and look forward to submitting our findings for peer review.*

Reviewer #3:

Thank you for the opportunity to peer-review this protocol. You make a strong case for the importance of conducting this scoping review. The protocol appears to be well-prepared and well-structured. I only have a few comments.

Introduction

Line 37, Page 7: «Evaluated» should be written «evaluate»

- *We have made this change to "evaluate".*

Line 5, page 8: The authors should consider rephrasing the third aim. As it stands now, it appears that the authors already make inferences based on the findings from the review.

- *Thank you for this comment. We have rephrased this to "Finally, based on our results, we will provide recommendations for use of the diagnostic criteria in pediatric clinical practice and research."*

Line 12, page 8: «This proposed scoping review is reported in accordance with the PRISMA-ScR guidelines» should be rewritten to «The proposed scoping review will be reported in accordance with the PRISMA-ScR guidelines».

- *The changes have been made, "This proposed scoping review will be reported in accordance with the PRISMA-ScR guidelines".*

Methods

If registered (e.g. in OSF), please provide the name of the registry and the registration number.

- *This scoping review is registered at OSF (https://osf.io/j8mp3/), and information is included in the manuscript.*

Line 18, page 8: The authors should provide a reference for limiting the pediatric population to up to 18 years.

- *We have now included the reference, to limit the pediatric population to the upper limit of 18 years.*

  Hardin AP, Hackell JM, Committee on Practice and Ambulatory Medicine, Simon GR, Boudreau AD, Baker CN, Barden III GA, Meade KE, Moore SB, Richerson J. Age limit of pediatrics. Pediatrics. 2017 Sep 1;140(3):e20172151.
